# Coping Styles and Stroke Knowledge Mediate the Effect of Social Support and Uncertainty Among Stroke Patients

**DOI:** 10.1093/geroni/igaa057.1463

**Published:** 2020-12-16

**Authors:** Humin Zhang, Jie Gao, Yuyan Sun, Ziting Han

**Affiliations:** 1 Xinxiang medical university, Xinxiang, Henan, China; 2 Xinxiang Medical University, Xinxiang, Henan, China; 3 Xinxiang Medical University, ZhengZhou, China

## Abstract

Objectives:This study aimed to investigate the mediating roles of coping styles and stroke knowledge between social support and uncertainty in illness among patients with primary stroke in China. Methods:The total of 204 Chinese primary stroke patients recruited using convenience sampling were asked to answer Mishel Uncertainty in Illness Scale for Adult(MUIS-A), Stroke Knowledge Questionnaire(SKQ), Social Support Rating Scale(SSRS), and Medical Coping Modes Questionnaires (MCMQ). Demographics characteristics of the patients were presented using descriptive statistics. We reported the relationship between the study variables using Pearson’s Correlation Coefficients. We performed structural equation modeling to estimate the mediator effect of coping styles and stroke knowledge between social support and uncertainty in illness.Results: The results showed that 92% of patients with primary stroke had moderate above level of uncertainty in illness, with a mean score 75.04 (SD=9.61).Uncertainty was positively associated with coping styles (r=0.232, P＜0.01), and negatively associated with social support(r=-0.237, P＜0.01) and stroke knowledge (r=-0.386, P＜0.01).The structural equation model indicated that the coping styles(19.8% of total effect) and stroke knowledge (38.5% of total effect)respectively acted as mediator role between social support and uncertainty in illness.Conclusions:Most patients with primary stroke present moderate above level of uncertainty in illness. stroke knowledge and coping styles were important mediating factors in the pathway between coping styles and uncertainty in illness. Our findings suggest the provision of stroke knowledge and training of coping styles for patients with primary stroke could alleviate their uncertainty in illness.

